# Supplementary material for: Anti-CD99 Antibody Therapy Triggers Macrophage-Dependent Ewing Cell Death In Vitro and Myeloid Cell Recruitment In Vivo
Source: Antibodies (Basel). 2024 Mar 18;13(1):24. doi: 10.3390/antib13010024 (PMC10967632; doi:10.3390/antib13010024)
Supplement: Supplementary file 1 [file antibodies-13-00024-s001.zip › antibodies-2797968-supplementary.pdf]

Supplementary Materials:

Methods

Phagemid panning for identification of anti-CD99 scFv's

Phagemid panning, utilizing a 27 billion member human single-chain variable fragment (scFv)-phage library (S. Mehta and W.A. Marasco), was performed for three rounds against immunotube-bound human CD99-Fc (G&P Biosciences). NUNC immunotubes were coated with soluble CD99 (10μg/mL) and incubated at 4°C overnight. They were subsequently washed with phosphate-buffered saline (PBS) and blocked with 4% milk/PBS at 37°C for 1-2 hours. During blocking, TG1 bacteria were grown in 2xYT media in a baked flask at 37°C, shaking at 250 rpm until growth was recorded at an OD600 of 0.5-0.7. The phagemid library was prepared for panning by diluting 5×10^{12} particles/mL in 2% milk/PBST (PBS-Tween), supplementing with isotype IgG to mitigate against binding to the Ig portion of CD99 Fc. Following immunotube blocking, tubes were washed with PBS, loaded with 1 mL of the phage sample, and incubated for 90 minutes on a rotating platform and then stationary, for 30 minutes, at room temperature. Immunotubes were washed and bound-phage eluted using triethylamine.

Eluted phage were neutralized with 1M Tris and used to infect TG1 cells. Serial dilutions of infected bacterial cultures were plated and grown overnight at 37°C, while the remainder of the culture was plated on a 15 cm bacterial dish and grown overnight at 30°C. The next morning, bacteria were harvested from the 15 cm dish and grown until an OD of 0.5-0.7 was achieved. A volume of 10 mL of this culture was infected with VCS-M13 helper phage at a multiplicity of infection (MOI) of 20. Bacterial cells were resuspended in 2xYT containing ampicillin and kanamycin, grown overnight, and centrifuged the following morning. Supernatant was collected and combined with 20% PEG/2.5 M NaCl in a 4:1 ratio, incubated on ice for 1 hour, and centrifuged. The resultant phage pellet was resuspended at 2mL and stored at 4°C. These rescued phage were then utilized for subsequent panning rounds against diminishing concentrations (lowest 1μg/mL) of immunotube-bound CD99.

Individual colonies from each round of panning were picked, cultured and analyzed for CD99 binding by ELISA. Positive binders were sequenced to determine the number of unique scFv binding clones. Each unique clone was assessed for binding to membrane-associated CD99 on patient-derived Ewing sarcoma cell lines by fluorescence-activated cell sorting (FACs). The resultant cell-binding scFv's were screened by ELISA against serial dilutions of soluble CD99 to identify the top three clones (NOA1, 2, and 3) with reproducible differential binding at concentrations of 0.5 μg/mL or less.

Molecular cloning and expression of anti-CD99 mAbs

The anti-CD99 heavy chain domains for each scFv were amplified and cloned into the MluI and NheI sites of a TCAE6-LL2 IgG expression vector, while scFv lambda light chains were cloned into the AvrII and HindIII sites. The final plasmids were transformed into E. Coli (strain DH5α). DNA was extracted from selected clones and sequenced to verify appropriate digestion and ligation. 293F cells were transfected with the anti-CD99 scFvFc expression plasmids and the cell culture supernatant was collected at day 7, filtered through a 45μm filter, and purified by Protein A affinity chromatography. SDS-PAGE gels were run to assess protein quality.

Primers utilized for molecular cloning

The following forward primers were used for heavy chain cloning: clones NOA1 and NOA2 forward: 5′- ATC GAC GCG TGT CCT GAG CCA GGT GCA GCT GGT G-3′ and NOA3 forward: 5′ - ATC GAC GCG TGT CCT GAG CGA GGT GCA GCT GGT G-3′. The following reverse primers were used for heavy chain cloning: clones NOA1 and NOA2 reverse: 5′ – CAT GCG CTA GCT GAA GAG ACG GTG ACC AT-3′ and clone NOA3 reverse: 5′ – CAT GCG CTA GCT GAG GAG ACG GTG ACC GT-3′. The following forward primers were used for light chain cloning: clone NOA1 forward: 5′ – ATC CCA AGC TTA AGC CAG TCT GTG CTG AC T-3′; clone NOA2 forward: 5′ – ATC CCA AGC TTA AGC CTG CCT GTG CTG ACT-3′; and clone NOA3 forward: 5′ – ATC CCA AGC TTA AGC TCC TAT GAG CTG ACT-3′. The reverse primers used for light chain cloning were as follows: clone NOA1 reverse: 5′- GCT GAC CTA GGA GGA CGG TGA CCT T-3′ and clones NOA2 and NOA3 reverse: 5′- GCT GAC CTA GGA GGA CGG TCA GCT T- 3′.

Flow cytometric analysis of CD47 expression

CD47 Ewing cell surface expression was analyzed by FACs for each Ewing sarcoma cell line at baseline and following treatment with NOA2 antibody. Ewing sarcoma cells of various lines (A673, TC32, CADO-ES1) were incubated in a FACs tube alone or with varying concentrations of NOA2 antibody (range: 0.125 to 2.0 μg/mL) for one hour and secondarily stained with 1.0 μg/mL of anti-CD47 FITC bound antibody (ThermoFisher Scientific). Percentage of CD47-expressing cells, as a function of pre-incubation NOA2 concentration, was tabulated.

Flow cytometric analysis of CD99 expression on PBMCs

A total of 1×10^6 unstained peripheral blood mononuclear cells were stained first with 2 μg/mL of NOA2 for one hour on ice, washed with autoMACs rinsing buffer (Militenyi Biotec) containing 1% BSA, and then incubated with 0.02 μL/mL of anti-human FITC-bound secondary (BD pharmingen, per manufacturers specifications). Cells were then washed and subsequently incubated with 0.02 μL/mL APC-bound anti-human CD33 (Biolegend, per manufacturers specifications). Cells were again washed three times in autoMACs rinsing buffer with 1% BSA and prepared for FACS.

Kinetic analysis of binding interactions between anti-CD99 and soluble CD99

Kinetic interactions between anti-CD99 antibody clones and soluble CD99-Fc (G&P Biosciences) were measured utilizing the Octet RED system (ForteBio). NOA2 and NOA3 were digested to form F(ab')2 fragments such that the soluble CD99-Fc could be immobilized on human Fc probes as the ligand, while the F(ab')2 fragments, at concentrates ranging from 0nM to 100nM, could serve as the analyte. NOA1 was omitted from this assay due to low-affinity binding across Ewing lines by FACs. ForteBio Octet software was utilized to analyze the data. Once binding curves were established, the experiment was repeated using analyte concentrations in a narrow range (10-15 nm for NOA2 and 10-25 nm for NOA3) so as to more accurately calculate each antibody K_D.

RESULTS

Isolation of CD99-specific scFv's from a phage display library and their genetic anaalysis

Two non-immune human scFv-phage display libraries containing 12 (Mehta I) and 15 (Mehta II) billion members were combined and incubated with immunotube-bound CD99-Fc. Bound scFvs were eluted from each panning round and screened by ELISA to confirm binding specificity. ELISA was first performed against an isotype-matched human monoclonal IgG1 to subtract out Fc-region binders, and subsequently against soluble CD99-Fc. Of the 50 resultant clones, DNA sequence analysis revealed that 10 were unique. Six of these phagemid scFvs bound to A673 Ewing sarcoma cells by FACs. Binding affinity for these six scFv's was assessed by ELISA with titration of soluble CD99-Fc concentrations. The top three clones, NOA1, 2, and 3 demonstrated saturable binding at concentrations of 0.125μg/mL or less (data not shown). These scFvs were later used for further studies.

The germline Ig gene segment usage and somatic hypermutation (SHM) are shown in **Supplementary Tables S1 and S2**. The NOA2 heavy chain is most closely aligned with the *03 allele of the IGHV5-51 germline gene, the *01 allele of the IGHD1-26 germline gene, and the *02 allele of the IGHJ3 germline segment. The heavy chain harbors seven single-nucleotide mutations (SNMs) including CDR1; however, CDR2 is in the germline configuration. The light chain utilizes IGLV5-37*01 and IGLJ3*02 germline genes and has six SNM.

Figure S1: Heavy and light chain sequences and comparative human germlines for the top three binding anti-CD99 scFv's (NOA1, 2, and 3). Red letters indicate single nucelotide mutations (SNMs).

```
                 FR1-IMGT                CDR1-IMGT     FR2-IMGT       CDR2-IMGT                   FR3-IMGT                             CDR3-IMGT           FR4-IMGT
                  (1-25)                  (26-33)      (34-50)         (51-58)                    (59-96)                              (97-113)           (114-125)
                 _________________________ ________  _________________ ________ ______________________________________  _________________  ___________
                 1        10        20        30         40        50          60        70        80        90         100       110         120
                 .........|.........|..... ....|...  .|....|.........| ........ .|.........|.........|.........|......  ...|.........|...  ......|.....

Germline
IGHV5-51*01/03   EVQLVQSGAEVKKPGESLKISCKGS GYSFTSYW  IGWVRQMPGKGLEWMGI IYPGDSDT RYSPSFQGQVTISADKSISTAYLQWSSLKASDTAMYYC ARPCSAGSCYSTDAFDI  WGQGTMVTVSS
NOA1 Clone       Q..........N............. ....P.H.  .........R....... ........ .......................LN.P........... .................  ...........

                                                                                                                      ARRANPDAFDI         WGQGTMVTVSS
NOA2 Clone       Q......P.............E.. .....N..  ................. ........ ....................S........Q...G.... ......G.....        ..........

                                                                                                                             ARHLRNFGDAFDI.     WGQGTMVTVSS
NOA3 Clone       ....................A.... .....SK...  .................. ........ ............V.T..................I... .............     .....T.....
```

```
                 FR1-IMGT                  CDR1-IMGT      FR2-IMGT    CDR2-IMGT          FR3-IMGT                      CDR3-IMGT    FR4-IMGT
                  (1-25)                    (26-31)        (32-48)    (49-51)             (52-87)                       (88-95)    (96-105)
             _________________________   ______   _________________  ___  ____________________________________   ________   __________
             1        10        20            30         40         50            60        70        80         90          100
             .........|.........|.....   ....|.   ........|........  .|.  .........|.........|.........|.......   ..|.....   ....|.....

Germline
IGLV3-1*01   SYELTQPPSVSVSPGQTASITCSGD   KLGDKY   ACWYQQKPGQSPVLVIY  QDS  KRPSGIPERFSGSNSGNTATLTISGTQAMDEADYYC   QAWDSSTA   FGTGTKVTVL
NOA1 Clone   QSV.............P........   ...NT.   VS...............  ..N  ....................................   ........   ..........

                                                                                                                           FGGGTKLTVL
NOA3 Clone   ..................R......   A.PKQ.   .Y...............  ...  ....................................   ........   ..........


                 FR1-IMGT                  CDR1-IMGT      FR2-IMGT        CDR2-IMGT            FR3-IMGT                   CDR3-IMGT   FR4-IMGT
                  (1-25)                    (26-34)        (35-51)         (52-58)              (59-96)                   (97-104)   (105-114)
             _________________________ _________ _________________  _______   ______________________________________ ________ __________
             1        10        20          30        40        50           60        70        80        90          100       110
             .........|.........|..... ....|.... .....|.........|.  .......   .|.........|.........|.........|...... ...|.... .....|....

Germline
IGLV5-37*01  QPVLTQPPSSSASPGESARLTCTLP SDINVGSYN IYWYQQKPGSPPRYLLY  YYSDSDK   GQGSGVPSRFSGSKDASANTGILLISGLQSEDEADYYC MIWPSNAS FGGGTKLTVL
NOA2 Clone   L........A............... ......... .................  .....N.   ...............T...................... ...H.S.W ..........
```

Table S1: Germline Segment Usage and Single Nucleotide Mutations for the top three binding anti-CD99 scFv's (NOA1, 2, and 3).

mAb	V_H	D_H	J_H	#SNM*	V_λ	J_λ	#SNM
NOA1	IGHV5-51*03	IGHD2-15*01	IGHJ3*02	7	IGLV3-1*01	IGLJ1*01	6
NOA2	IGHV5-51*03	IGHD1-26*01	IGHJ3*02	7	IGLV5-37*01	IGLJ3*02	6
NOA3	IGHV5-51*01	IGHD3-16*01	IGHJ3*02	7	IGLV3-1*01	IGLJ3*02	6

H: heavy chain, λ: lambda (light) chain, #SNM: number of single nucleotide mutations
*SNM tabulation excludes the 5′ end mutations as these are attributed to substitutions introduced during priming.

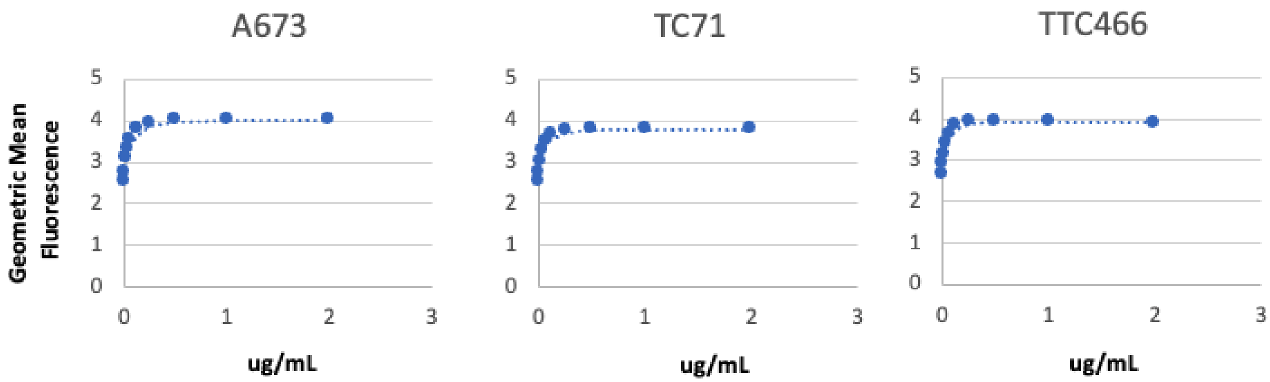

Supplementary Figure S2: Repeat FACS analysis to depict the geometric mean fluorescence of A673, TC71 and TTC466.

Ewing cell CD47 expression following incubation with NOA2

We next investigated whether CD47, a surface molecule known to inhibit phagocytosis was involved in the differential ADCP effects among the cell lines noted above. FACS analysis was performed as a function of pre-treatment with various concentrations of NOA2. As can be seen in **Supplementary Figure S1**, while lower concentrations of NOA2 consistently showed a decrease in CD47 expression in all three cell lines, A673 cells showed circa 35% upregulation in expression at the highest concentrations of NOA2 compared to 18% and 0% increased in expression in CADO and TC32 cells. It is unclear if this modest increase in CD47 expression could account for the lack of ADCP in A673 cells.

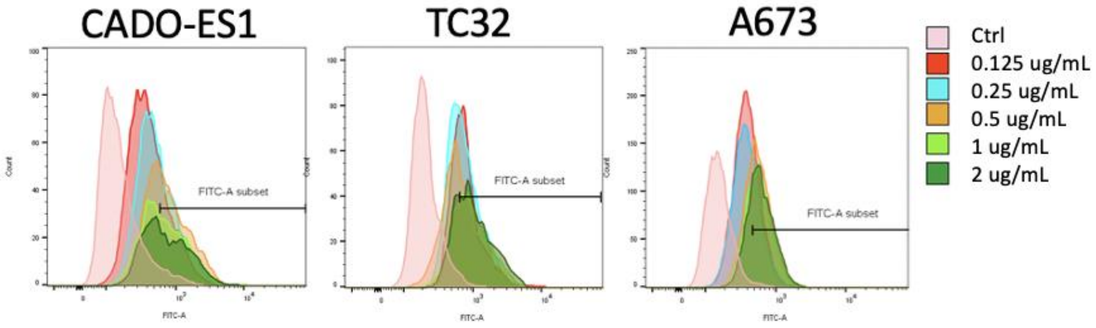

		Ctrl (-)	0	0.125	0.25	0.5	1	2
CADO-ES1	Anti-CD47-FITC (1 ug/mL)	5.43	46.1%	25.9%	38.4%	56.7%	52.5%	54.5%
TC32	Anti-CD47-FITC (1 ug/mL)	2.42	74.3%	63.0%	55.9%	49.4%	71.1%	74.5%
A673	Anti-CD47-FITC (1 ug/mL)	1.88	52.8%	30.8%	30.4%	64.9%	67.0%	72.0%
		Cells alone	Cells incubated with 1 ug/mL of anti-CD47					

Supplementary Figure S3: CD47 in CADO-ES1, TC32, and A673 following pre-treatment with NOA2. All three cell lines demonstrate a modest increase in CD47 expression following pre-treatment with varying doses of NOA2.

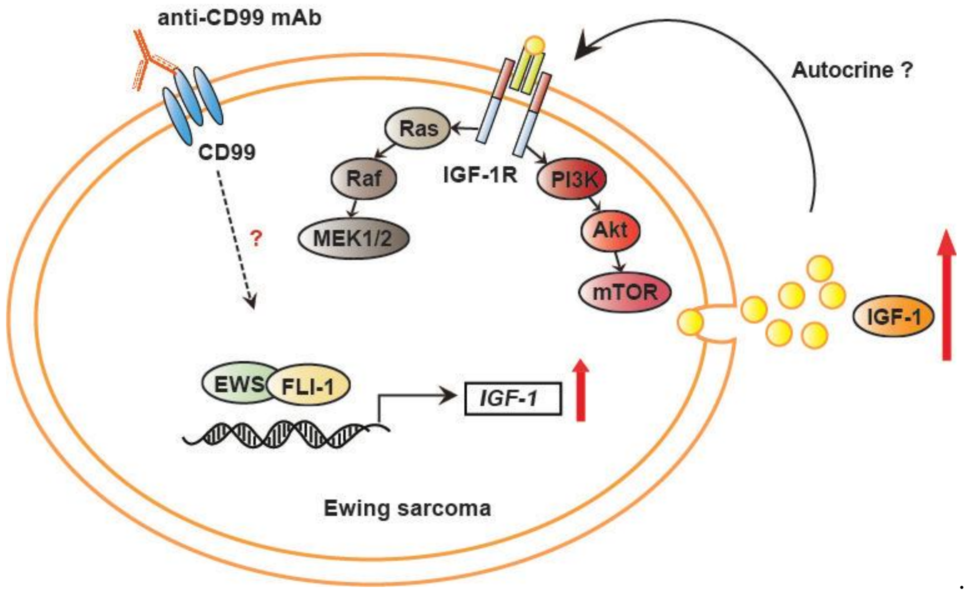

Supplementary Figure S4: The proposed downstream effects of NOA2 binding to membranous CD99 on Ewing sarcoma cells resulting in increased IGF-1 transcription and secretion which then acts to further upregulate the IGF-1/IGF-1R pathway through an autocrine loop.

Monocyte expression of CD99 as detected by NOA2

PBMCs were gated by side and forward scatter to separate monocytes and lymphocytes by size. There was non-specific binding of anti-human FITC-bound secondary to monocytes which was accounted for by gating. Nearly 70% of CD33-positive monocytes demonstrated circa 3-fold binding of NOA2 (**Supplementary Figure S3**).

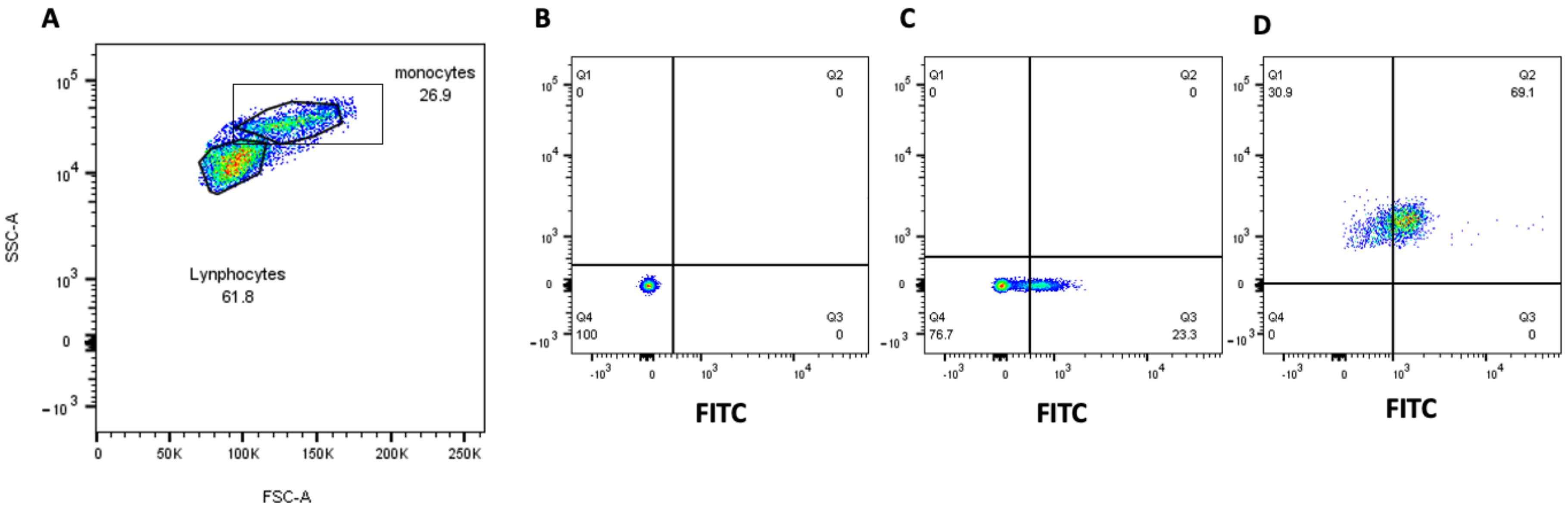

Supplementary Figure S5: Cell surface expression of CD99, detected by NOA2 binding, on monocytes derived from human peripheral blood mononuclear cells. (Panel A) demonstrates PBMC separated into lymphocyte and monocyte populations (depicted within the superimposed square) based on side and forward scatter. Monocytes were unstained (Panel B) and incubated with anti-human FITC-bound secondary (Panel C). Panel C shows non-specific binding of the anti-human FITC-bound secondary to monocytes, which is accounted for in gating of cells in Panel D. The population of CD33+ cells that express CD99 (by NOA2 binding) are depicted in Panel D
